# Comparison of the Methods of Removal of Cataract with and without Iridectomy

**Published:** 1887-05

**Authors:** H. Knapp

**Affiliations:** New York


COMPARISON OF THE METHODS OF REMOVAL OF CATARACT WITH AND WITHOUT IRIDECTOMY. BY DR. H. KNAPP, *of New York.*

If we compare the simple extraction with the extraction combined with iridectomy, we find as advantages of the former the following:

1. It preserves the natural appearance of the eye.

2. The acuteness of vision, other things being equal, is greater.

3. Eccentric vision and "orientation" (correct localization of objects in the visual field) are much better, adding a great deal to the comfort and safety of the patient.

4. Parts in direct connection with the ciliary body, such as shreds of the capsule and iris, are not so liable to be locked up in the wound and thus transmit morbid conditions to the most vulnerable part of the eye, the ciliary body.

5. It may not necessitate so many after-operations.

As *disadvantages* may be mentioned:

1. The technique of the operation is more difficult in all its parts: (*a*) The section must be larger, to let the lens pass through an aperture the size of which is diminished by the iris lying in it; it must be more accurate to secure coaptation, and it must be more rapidly performed in order to prevent the iris from falling before the knife. (*b*) The opening of the capsule requires a deeper introduction of the cystitome into the anterior chamber. (*c*) The expulsion of the lens is more difficult, and (*d*) the cleansing of the pupillary area is much more troublesome than in the combined extraction.

2. Prolapse of iris and posterior synechiæ are more numerous.

3. It requires a quieter and more manageable patient during and after the operation than is needed in the combined extraction.

4. It is not applicable to all patients, whereas combined extraction can be used as a general method.—*Archives of Ophthalmology for March, 1887.*

